# Innovative Strategy for 3D Transfection of Primary Human Stem Cells with BMP-2 Expressing Plasmid DNA: A Clinically Translatable Strategy for Ex Vivo Gene Therapy

**DOI:** 10.3390/ijms20010056

**Published:** 2018-12-23

**Authors:** Maruthibabu Paidikondala, Sandeep Kadekar, Oommen P. Varghese

**Affiliations:** Translational Chemical Biology Laboratory, Polymer Chemistry Division, Department of Chemistry–Ångström Laboratory, Uppsala University, 751 21 Uppsala, Sweden; maruthibabu.paidikondala@kemi.uu.se (M.P.); sandeep.kadekar@kemi.uu.se (S.K.)

**Keywords:** hydrogel, DNA, transfection, ex vivo, hyaluronic acid

## Abstract

Ex vivo gene therapy offers enormous potential for cell-based therapies, however, cumbersome in vitro cell culture conditions have limited its use in clinical practice. We have optimized an innovative strategy for the transient transfection of bone morphogenetic protein-2 (BMP-2) expressing plasmids in suspended human stem cells within 5-min that enables efficient loading of the transfected cells into a 3D hydrogel system. Such a short incubation time for lipid-based DNA nanoparticles (lipoplexes) reduces cytotoxicity and at the same time reduces the processing time for cells to be transplanted. The encapsulated human mesenchymal stromal/stem cells (hMSCs) transfected with BMP-2 plasmid demonstrated high expression of an osteogenic transcription factor, namely RUNX2, but not the chondrogenic factor (SOX9), within the first three days. This activation was also reflected in the 7-day and 21-day experiment, which clearly indicated the induction of osteogenesis but not chondrogenesis. We believe our transient transfection method demonstrated in primary MSCs can be adapted for other therapeutic genes for different cell-based therapeutic applications.

## 1. Introduction

Gene therapy holds great promise to treat a variety of human conditions and has been clinically evaluated for treating several diseases such as cancer, cardiovascular disease and immunological conditions, as well as for developing drugs for regenerative medicine [1,2]. Regenerative medicine applications generally require local delivery of proteins, genes or cells, which is carried out using 3D scaffolds [3]. We have recently developed injectable hydrogels that can sequester recombinant human bone morphogenetic protein-2 (rhBMP-2) and promote bone formation in vivo, mimicking the natural behavior of the extracellular matrix [4]. Though this is very promising, delivering osteogenic genes locally at the defect site, instead of the protein, will act as a reservoir for the protein that will reduce the systemic toxicity and lower the need for supra-physiological dosages of therapeutic proteins [5]. Therapeutic proteins such as rhBMP-2 are also prone to aggregation at physiological pH, limiting their scope for clinical applications [6]. Thus, innovative strategies for designing gene-activated 3D matrices (GAMs) that work as a mini-cellular factory producing therapeutic proteins in situ is highly desired. The GAMs are generally composed of synthetic or biopolymers containing DNA nanoplexes [7], nanoparticles (e.g., nanohydroxyapatite) [8,9], hydrogels containing cationic groups [10] or cationic polymers such as chitosan [9,11]. They stabilize plasmid DNA and promote gene delivery to the cells that are in the vicinity. Though such a strategy has been shown to stimulate osteogenesis and bone repair at the defect site [7,8,9,12], the reproducibility of such methods are dependent on several critical factors that include the number of cells that are transfected, recruitment of desired cells into the scaffold for cell-free strategies [9], stability of plasmid DNA within the biological milieu, the retention or release of the plasmid from the scaffold, and the wound environment. Thus, instead of in vivo gene delivery, ex vivo gene delivery strategies have great potential provided that they can be achieved efficiently without inducing toxicity and can be directly applied with patient-derived primary cells without the complicated in vitro cell culture procedure.

Intracellular delivery of plasmid DNA has been the center of scientific investigation for over three decades. Large anionic drugs such as plasmid DNA cannot penetrate the plasma barrier, even though naked DNA has been shown to enable some level of transgene expression in vivo [13]. The transfection efficiency of such a method is, however, very limited. Employing viral vectors to deliver such molecules has been the most successful strategy so far, albeit, the immunological aspects of such vectors and the risk of insertional mutagenesis are a major concern [14]. Recently, the first gene-based drug, Glybera, delivered through a viral vector, has been approved in Europe for treating patients with hereditary lipoprotein lipase deficiency (LPLD) [15]. The extremely high cost of this drug has limited its scope and it has recently been withdrawn from the market. Recently another gene-based drug, Invossa^TM^ (ClinicalTrials.gov Identifier: NCT03203330), has been approved in South Korea for treating arthritis. It is interesting to note that all the clinically approved gene-based drugs so far involve a viral-based formulation, as this strategy allows endogenous expression of clinically beneficial amounts of proteins in a sustained manner. The non-viral vectors, which are usually based on cationic polymers or lipids that electrostatically bind plasmid DNA, are not as efficient as viral vectors [16]. Such cationic polymers were also shown to induce toxicity and are believed to bind cell-surface glycosaminoglycans (GAGs) such as heparan sulfate that alter cationic nanoparticle stability and endocytosis [17]. The key hurdle for such formulations is the endosomal escape of the cargo molecules and the process of nuclear transport to initiate transcription. We have recently shown that cationic DNA nanoparticles coated with anionic GAGs such as chondroitin sulfate significantly improve transfection efficiency by controlling plasmid uptake and release from the endosomes [18].

**Scheme 1:**
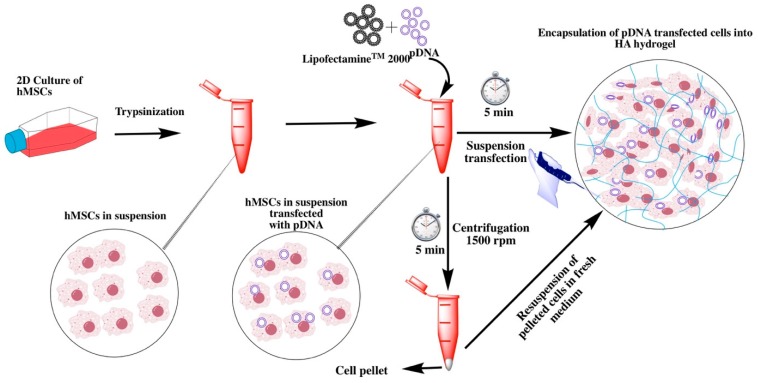
Schematic representation of suspension transfection of hMSCs followed by encapsulation into a hyaluronic acid (HA) hydrogel.

For clinical applications in vivo, gene therapy is more ideal than ex vivo transfection. However, in-vivo gene delivery approaches are often laden with problems, such as a lack of efficient targeted delivery vehicles that do not exert toxicity, side effects exerted by non-specific targeting or leakage of the therapeutic gene to the non-targeted tissues, and enzymatic degradation of the delivered gene. Therefore, cell-based therapies powered with ex vivo gene transfer have emerged as an alternative. However, ex vivo transformation of cells is associated with expensive manufacturing conditions and is generally time-consuming. Moreover, toxicity posed by the transfection reagent and efficiency of gene delivery are other issues impeding ex-vivo application in the clinical scenario. In comparison, direct injection of plasmid DNA with or without a specific delivery vehicle in vivo is relatively less complicated, however, for effective tissue regeneration, a significant number of host cells need to be transfected. The toxicity of the transfection reagent should be mitigated if the DNA nanoplexes are directly injected or delivered using a 3D scaffold. Ex vivo manipulation of the cells, on the other hand, will allow efficient transfection, as well as permit possibilities to remove excessive reagent. We therefore decided to investigate if it is possible to transfect cells with BMP-2 expressing plasmid in suspension conditions for a short incubation time. Traditionally, suspension transfection is carried out by incubating non-adherent cells and lipoplexes for at least 5–6 h [19]. Since cationic lipid-based reagents are known to promote cytosolic delivery of the cargo molecules within 5–15 min of endocytosis, we envisioned that a short incubation time with cells and lipoplexes could deliver the cargo molecules to the cytosol [20]. For this purpose, we selected Lipofectamine^TM^ 2000 as the transfection reagent. The majority of the cargo molecule that is endocytosed usually does not escape the endosomal compartment except some amount that is released by membrane fusion. We anticipated that such a quick transfection method (Scheme 1) could be advantageous as it could allow transfection of patient-derived cells without much manipulation.

## 2. Results and Discussion

In order to investigate suspension transfection efficiency followed by implantation in a biocompatible 3D scaffold, we performed transfection experiments with BMP-2 expressing plasmid in human bone marrow-derived mesenchymal stromal/stem cells (MSCs) and transplanted them in a hydrazone crosslinked hyaluronic acid (HA) hydrogel that was previously optimized in our laboratory. Briefly, hydrogels were obtained by mixing aldehyde-modified HA [21], and hydrazide-modified polyvinyl alcohol [22]. Since such hydrogels mimic the natural extracellular matrix (ECM), they could be directly applied to clinical applications as previously demonstrated by our group [23]. To evaluate the transfection efficiency, we first performed experiments with adherent and suspension cells using Lipofectamine^TM^ 2000 as the transfection reagent and luciferase-expressing plasmid DNA as the reporter. We optimized the transfection experiments under suspension conditions by incubating the lipoplexes (lipid-DNA complex) with MSCs for 5 and 10 min and compared the transfection efficiency with adherent conditions. Interestingly, ONE-Glo™ Luciferase Assay of the cell lysate after 48 h of transfection indicated that both suspension and adherent cells were equally efficient. We did not observe any significant difference between 5 and 10 min incubations, indicating that cytosolic delivery of the cargo molecules happened in the first couple of minutes (Figure S1). We, therefore, continued further experiments with 5-min incubation and BMP-2 expressing plasmid DNA.

To identify the best in vivo translatable gene transfection condition, we tested three different experimental conditions to evaluate BMP-2 plasmid expression in 2D namely, adherent, suspension, and suspension followed by centrifugation. For the adherent condition, the bone marrow-derived stem cells were plated 24 h prior to the transfection experiment, whereas, for suspension and suspension followed by the centrifugation conditions, cells were transfected in suspension while they were being plated. This implies that the suspension condition takes 24 h less time than the adherent transfection condition. An additional centrifugation step was employed in a separate group in order to evaluate the transfection efficiency after removing excess reagent or lipoplexes.

**Figure 1:**
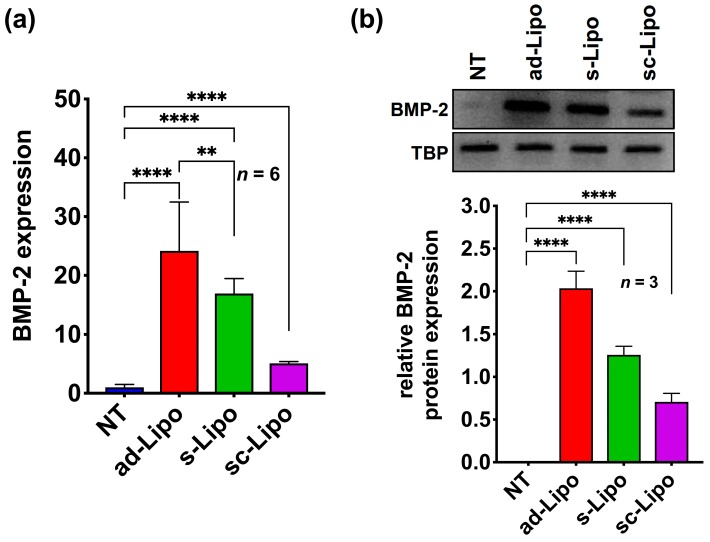
In vitro analysis of BMP-2 expressing plasmid DNA transfected in hMSCs in 2D cell culture. (**a**) Quantitative RT-PCR results indicating the expression levels of BMP-2 mRNA in hMSCs after 48 h of transfection. TBP was employed as an internal control to normalize BMP-2 mRNA expression. Gene expression values are stated as fold change relative to controls. Mean values ± SD of 2 independent experiments done in triplicates (*n* = 6) are shown. Statistical analysis was performed using one-way ANOVA with Bonferroni’s multiple comparison corrections (** *p* < 0.001, **** *p* < 0.00001). (**b**) Western blot analysis indicating BMP-2 protein levels after 72 h of transfection. BMP-2 and TBP-specific bands were quantified by ImageJ software and BMP-2 protein expression levels were normalized to TBP values. Mean values ± SD of three independent experiments are shown. The abbreviation NT = non-treated cells; ad-Lipo = Lipofectamine^TM^ 2000 assisted transfection with adherent cells; s-Lipo = Lipofectamine^TM^ 2000 assisted transfection of cells in suspension; sc-Lipo = Lipofectamine^TM^ 2000 assisted transfection of cells in suspension followed by centrifugation; TBP = TATA-binding protein (**** *p* < 0.0001).

To evaluate the transfection efficiency, we performed real-time PCR (qRT-PCR) experiments after 48 h of transfection, which indicated that Lipofectamine^TM^ 2000-based transfection of a BMP-2 plasmid DNA in 2D cell culture could successfully deliver the target gene into human MSCs in all transfection conditions tested (adherent, suspension, suspension followed by centrifugation). However, BMP-2 expression varied between different groups tested. Most notably, the adherent condition demonstrated a relatively high expression of BMP-2 compared to the suspension condition, whereas, the centrifugation condition significantly reduced the expression of BMP-2 (Figure 1a). This indicates that under suspension conditions the uptake is slower and the removal of lipoplexes from solution affects transfection levels. We further examined BMP-2 expression at the translational level using western blot analysis after 72 h of transfection (Figure 1b). Taken together, these results confirm that both adherent and suspension conditions are efficient enough to express significant levels of BMP-2 protein in hMSCs.

**Figure 2:**
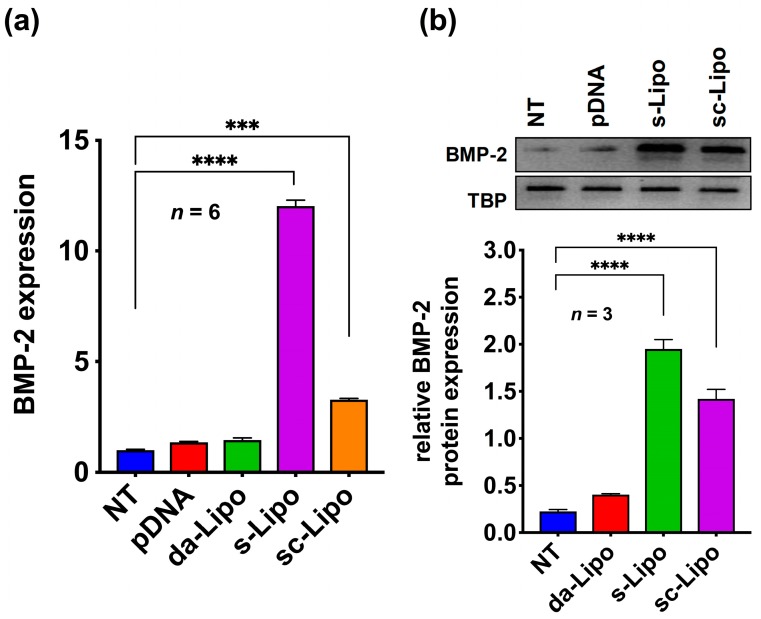
In vitro analysis of transfection of BMP-2 expressing plasmid DNA in hMSCs followed by encapsulation in HA-hydrogels. (**a**) Quantitative RT-PCR results indicating the expression levels of BMP-2 mRNA in hMSCs after 48 h of transfection in 3D. TBP was employed as an internal control to normalize BMP-2 mRNA expression. Gene expression values are stated as fold change relative to controls. Mean values ± SD of 2 independent experiments done in triplicates (*n* = 6) are shown. Gene expression values are stated as fold change relative to controls. Statistical analysis was performed using one-way ANOVA with Bonferroni’s multiple comparison corrections (*** *p* < 0.0001, **** *p* < 0.00001). (**b**) Western blot analysis indicating BMP-2 protein levels after 72 h of transfection. BMP-2 and TBP-specific bands were quantified by ImageJ software and BMP-2 protein expression levels were normalized to TBP values. Mean values ± SD of 3 independent experiments are shown.The abbreviation NT = non-treated cells; pDNA = plasmid DNA alone; da-Lipo = direct addition of lipoplexes to MSC encapsulated in HA-hydrogel; s-Lipo = Lipofectamine^TM^ 2000 assisted transfection of cells in suspension followed by encapsulation in HA-hydrogel; sc-Lipo = Lipofectamine^TM^ 2000 assisted transfection of cells in suspension followed by centrifugation and subsequent encapsulation in HA-hydrogel; TBP = TATA-binding protein (**** *p* < 0.00001).

Encouraged by the transfection efficiency in the 2D cell culture condition, we decided to replicate the results in a 3D hydrogel system. We repeated the suspension transfection experiments with 5-min incubation as discussed above (suspension and suspension followed by centrifugation) and loaded cells inside the HA hydrogel. As a control, we loaded plasmid DNA alone (without forming lipoplexes) and lipoplexes into the 3D hydrogel containing hMSCs. Quantitative RT-PCR analysis of different groups revealed that the Lipofectamine^TM^ 2000 assisted suspension transfection in HA hydrogels yields the maximum expression of the BMP-2 protein. However, a relatively lower expression of BMP-2 plasmid was observed in the case of suspension transfections followed by centrifugation. Moreover, direct addition of BMP-2 plasmid DNA or addition of lipoplexes did not show any expression of the protein (Figure 2a). These results were further corroborated by Western blot experiments, which indicated that there is a clear advantage of suspension transfection in 3D cell culture over the standard method usually described in gene activated matrices (Figure 2b) [24].

**Figure 3:**
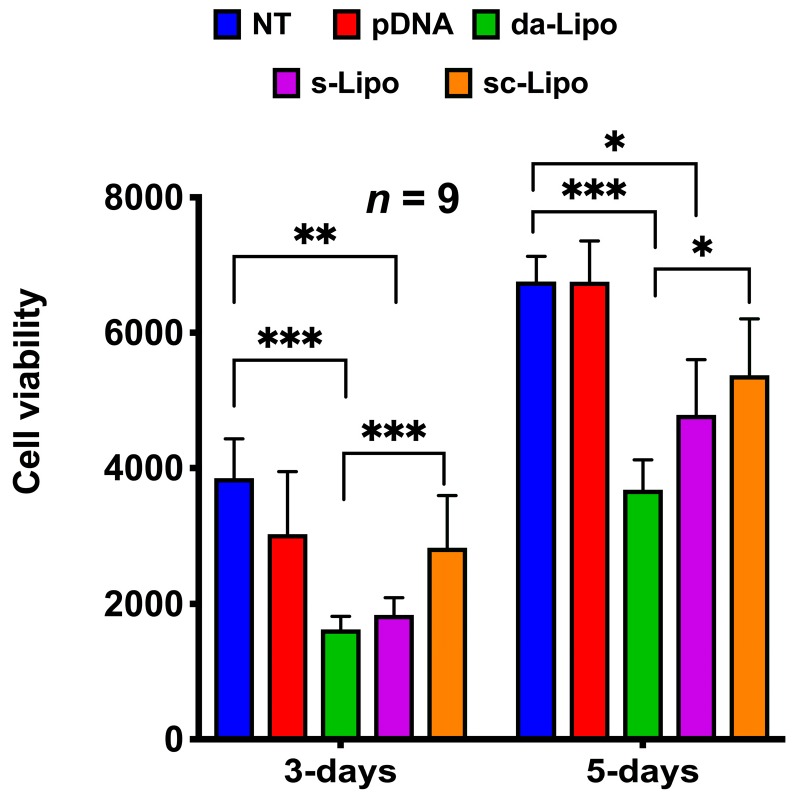
Cytotoxicity studies of hMSCs transfected with BMP-2 plasmid encapsulated in HA hydrogels after 3- and 5-days. The abbreviation NT = non-treated cells; pDNA = plasmid DNA alone; da-Lipo = direct addition of lipoplexes to MSC encapsulated in HA-hydrogel; s-Lipo = Lipofectamine^TM^ 2000 assisted transfection of cells in suspension followed by encapsulation in HA-hydrogel; sc-Lipo = Lipofectamine^TM^ 2000 assisted transfection of cells in suspension followed by centrifugation and subsequent encapsulation in HA-hydrogel. Mean values ± SD of 3 independent experiments done in triplicates (*n = 9)* are shown. Statistical analysis was performed using one-way ANOVA with Bonferroni’s multiple comparison corrections (* *p* < 0.01, ** *p* < 0.001, *** *p* < 0.0001).

Biocompatibility is an important requirement for the safe clinical translation of hydrogels with osteogenic potential that can efficiently regenerate tissues, such as bone. Such hydrogels should also not exert any toxicity, which in turn could compromise the repair process. We, therefore, assessed the cell viability of hMSCs embedded in HA hydrogels using Presto Blue assay at 3- and 5-days post-transfection (Figure 3). For this analysis, we performed suspension transfection experiments for 5-min as discussed above (suspension and suspension followed by centrifugation) and loaded transfected cells inside the HA hydrogel. As a control, we loaded plasmid DNA alone (without forming lipoplexes) and lipoplexes into the 3D hydrogel containing hMSCs. The cell viability was measured with respect to the non-treated (NT) hMSCs in the hydrogel. Interestingly, these experiments indicated that the transfection reagent induced cytotoxicity to MSCs, where direct addition of lipoplexes to hydrogel was the most toxic, while suspension transfection followed by centrifugation was the least toxic. Though there is a clear correlation between centrifugation and reduced toxicity, our experimental condition may not be efficient enough to remove the excess reagent completely. Nevertheless, our experiments indicate that the toxic effects of the reagents diminish over time, as the cytotoxicity after 5-days of experiment appears to be less than that of the 3-days post-transfection experiment.

Encouraged by the results obtained from 2D and 3D transfection experiments in human MSCs, we further investigated if the overexpression of BMP-2 could lead to osteogenesis in the targeted cells encapsulated in HA hydrogel. Evaluating the expression levels of several important early and late differentiation markers could validate BMP-2-mediated osteoblastic differentiation of human MSCs. One of the most important early markers for osteogenesis is the activation of the transcription factor RUNX2 that binds to specific DNA sequences and thereby plays a crucial role in modulating genes related to bone formation [25,26]. The non-canonical BMP signaling also results in over-expression of alkaline phosphatase (ALP) indicating a pre-osteoblast phenotype. Complete differentiation of hMSCs to mature osteoblasts could be determined by measuring the late osteogenic markers such as osteocalcin (OCN), and osteopontin (OPN) [27]. Osteogenic differentiation should also lead to bone-specific ECM production with the expression of collagen type-I (Col1), a hallmark of mature osteoblasts [28]. Since endochondral ossification is generally observed during the bone healing process, the expression pattern of the early chondrogenic marker SOX9 could indicate the mechanism of bone formation. This could also be validated by quantifying the expression of the chondrogenic ECM component collagen II (Col2A1) [29].

**Figure 4:**
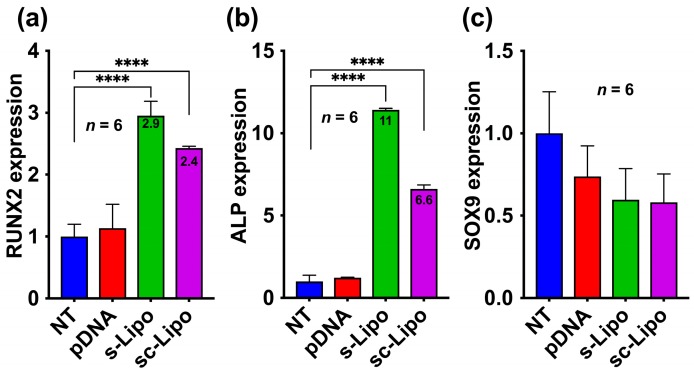
Quantitative RT-PCR of hMSCs transfected with BMP-2 plasmid followed by encapsulation in HA hydrogels after 7-days. (**a**) Expression profile of early osteogenic markers, RUNX2, and (**b**) ALP after 7-days of the experiment. (**c**) Expression profile of chondrogenic markers after 7-days of the experiment. Mean values ± SD of 2 independent experiments done in triplicates (*n* = 6) are shown. TBP was employed as an internal control to normalize BMP-2 mRNA expression. Gene expression values are stated as fold change relative to controls. Statistical analysis was performed using one-way ANOVA with Bonferroni’s multiple comparison corrections (**** *p* < 0.00001). The abbreviation NT = non-treated cells; pDNA = plasmid DNA alone; s-Lipo = Lipofectamine^TM^ 2000 assisted transfection of cells in suspension followed by encapsulation in HA-hydrogel; sc-Lipo = Lipofectamine^TM^ 2000 assisted transfection of cells in suspension followed by centrifugation and subsequent encapsulation in HA-hydrogel.

In order to assess the osteogenic or chondrogenic differentiation of human MSCs that were transfected with the BMP-2 plasmid in 3D, we examined both early and late differentiation markers. We first evaluated the expression of RUNX2 and SOX9 at 7-days post-transfection through quantitative RT-PCR analysis. Our results indicate that the suspension transfection of human MSCs and consequent loading in HA hydrogels successfully led to the overexpression of RUNX2 and ALP but not SOX9 (Figure 4a–c). This indicates that bone formation with such stem cell-loaded hydrogels will lead to direct ossification and will not follow endochondral ossification. Interestingly, suspension transfection followed by centrifugation also demonstrated an increase in osteogenic differentiation, unlike what was anticipated from the 3-day experiments. This is presumably due to the fact that osteogenic differentiation of MSCs has a paracrine effect that directs differentiation of other surrounding cells. Such effects are more pronounced in 3D as the cell lays down peri-cellular matrix within hours that sequesters cell-secreted factors within the matrix [30].

**Figure 5:**
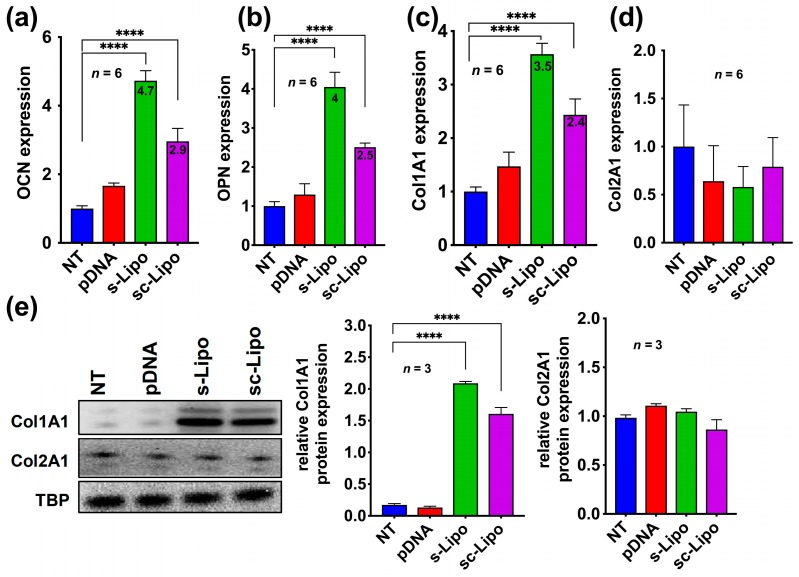
In vitro evaluation of osteogenic differentiation of hMSCs transfected with BMP-2 plasmid followed by encapsulation in HA hydrogels for 21-days. (**a**) Quantitative RT-PCR analysis indicating the expression profiles of late osteogenic markers, osteocalcin (OCN) (**b**) osteopontin (OPN) and (**c**) collagen-1 (Col1A1) after 21-days of the experiment. (**d**) Expression profile of chondrogenic marker collagen-2 (Col2A1) after 21-days of the experiment. TBP was employed as an internal control to normalize BMP-2 mRNA expression. Gene expression values are stated as fold change relative to controls. Mean values ± SD of 2 independent experiments done in triplicates (*n* = 6) are shown (**e**) Western blot analysis indicating the expression of Col1A1 and Col2A1 as a result of transfection with BMP-2 in 3D. Col1A1, Col2A1, and TBP-specific bands were quantified by ImageJ software. Both Col1A1, and Col2A1 protein expression levels were normalized to TBP values. Mean values ± SD of three independent experiments are shown. Statistical analysis was performed using one-way ANOVA with Bonferroni’s multiple comparison corrections (**** *p* < 0.00001). The abbreviation NT = non-treated cells; pDNA = plasmid DNA alone; s-Lipo = Lipofectamine^TM^ 2000 assisted transfection of cells in suspension followed by encapsulation in HA-hydrogel; sc-Lipo = Lipofectamine^TM^ 2000 assisted transfection of cells in suspension followed by centrifugation and subsequent encapsulation in HA-hydrogel; TBP = TATA-binding protein.

To further validate our results, we evaluated the gene induction after 21-days of transfection. We specifically assessed the expression of key late markers of osteoblast differentiation such as OCN, OPN, and Col1A1 by qRT-PCR. For comparison, we also determined the expression of late chondrogenic marker Col2A1. These experiments indicate that both suspension transfection and suspension transfection followed by centrifugation leads to a significant upregulation of late osteoblastic genes OCN, OPN, Col1A1 but not chondroblastic genes Col2A1 after 21-days post-transfection, corroborating with results observed after 7-days experiments (Figure 5a–d). These results were further validated by Western blot analysis, which demonstrated overexpression of Col1A1 but not Col2A1 at the protein level (Figure 5e).

## 3. Materials and Methods

### 3.1. Cell Culture

Human bone marrow-derived MSCs were maintained in complete DMEM medium (Cat#10567014; Gibco-Invitrogen, Carlsbad, CA, USA) with 10% hMSC grade fetal bovine serum (FBS; Cat# 12662029; Gibco), 1% penicillin and streptomycin (Pen-Strep DE17-602E, Lonza, Basel, Switzerland) and 5 ng/mL recombinant human fibroblast growth factor (FGF-2) (Cat# 100-18B; PEPROTECH, Rocky Hill, NJ, USA). Cells were incubated at 37 °C and 5% CO_2_ and the medium was refreshed every second day.

### 3.2. Adherent, Suspension, and Suspension Followed by Centrifugation Conditions in 2D

For adherent conditions one day before the experiment, 35000 cells per well were plated in a 24-well cell culture plate. Lipofectamine^TM^ 2000 Transfection Reagent kit and protocol from Thermofisher (Rockford, IL, USA) was used for the transfection of 100 ng of plasmid DNA (EF1alpha-BMP2 [31]) per well. For suspension condition, we added Lipofectamine^TM^ 2000 and pDNA complex in opti-MEM medium to the tube containing 35,000 cells in cell culture medium with heat-inactivated FBS and incubated for 10 min and transferred to the respective wells. For suspension followed by centrifugation conditions, we mixed lipofectamine™ 2000 and pDNA complex in opti-MEM medium to the tube containing 35,000 cells in cell culture medium with heat-inactivated 10% FBS and collected cells after brief centrifugation for 5 min at 1600 rpm and the supernatant was aspirated. Thereafter, cells were resuspended in cell culture medium and transferred to the respective wells. All experiments were done in triplicates and repeated twice. Statistical analysis was done using one-way ANOVA with Bonferroni’s multiple comparison corrections (* *p* < 0.00001).

### 3.3. pLuc Expression Studies in 2D

In order to assess the shortest possible time for Lipofectamine™ 2000 based transfection in 2D, we tested 5 min, and 10 min incubation times in three different conditions: adherent, suspension, and suspension followed by centrifugation as explained above. Two days post-transfection of luciferase-expressing pGL-3 plasmid (#Cat E1751; from Promega, Madison, WI, USA), ONE-Glo™ Luciferase Assay System and protocol was used to measure the luciferase expression in tested wells in quadruplicates and compared with non-treated (NT) control. All experiments were done in triplicates and repeated twice. Statistical analysis was done using one-way ANOVA with Bonferroni’s multiple comparison corrections (* *p* < 0.0001).

### 3.4. Direct Addition, Suspension, Suspension Followed by Centrifugation Conditions in 3D

Hyaluronic acid (HA) hydrogel having 1.8% (*w*/*v*) of solid content was prepared as described earlier with ≈10% aldehyde modified HA (HA-CHO) [21] and ≈20% hydrazide modified polyvinyl alcohol (PVA-hydrazide) [22]. Briefly, 16 mg/mL of aldehyde-modified HA was dissolved in cell culture grade sterile PBS without Ca and Mg, while 3 mg/mL of PVA-hydrazide was dissolved in DMEM medium without FBS and sterile filtered with Acrodisc^®^ Syringe Filter 0.8 μM Supor^®^ Membrane (sterile) low protein binding Non-Pyrogenic (PN 4608) from PALL Life Sciences (Hampshire, UK). On the other hand, cells were prepared from 2D culture through trypsinization. Thereafter, nearly 2.5 million cells were suspended in 1 mL of PVA-hydrazide (3 mg/mL) solution. In order to make a 200 μL hydrogel, 120 μL of 16 mg/mL HA-aldehyde solution in PBS was mixed with 80 μL of 3 mg/mL PVA-hydrazide with 200,000 cells in cell culture medium. For direct addition, BMP2-pDNA-HA hydrogels were prepared by adding 500 ng BMP2-pDNA to 120 μL HA-CHO solution (16 mg/mL) made in PBS. To make a gel, HA-CHO+BMP2-pDNA and PVA-hydrazide solution containing cells were mixed together and placed in a 24-well culture plate and incubated for 1 h for gelation at 37 °C and 5% CO_2_. After gelation, 500 μL of the complete DMEM medium was added and incubated at 37 °C for 72 h.

For the suspension transfection condition in hydrogels, 500 ng of BMP2-pDNA, 1.5 μL of Lipofectamine^TM^ 2000 was added to 120 μL HA-aldehyde solution (16 mg/mL) made in cell culture grade sterile PBS without Ca and Mg. To this complex, we have added 80 μL of 3 mg/mL PVA-hydrazide prepared in cell culture medium with 200,000 cells in a 24-well cell culture plate. For suspension followed by the centrifugation condition, we have pre-transfected hMSCs as explained above. Later, we performed centrifugation at 1600 rpm for 5 min, discarded the supernatant to remove any extra Lipofectamine^TM^ 2000 reagent and collected cells. Thereafter, these cells were encapsulated into HA-PVA hydrogels. All experiments were done in triplicates and repeated twice. Statistical analysis was done using one-way ANOVA with Bonferroni’s multiple comparison corrections (* *p* < 0.0001).

### 3.5. Cytotoxicity Studies in 3D

Different groups of HA hydrogels (100,000 cells in 100 μL gel) were prepared in triplicates as explained above in a black 96-well plate with a transparent bottom. After 3-days and 5-days, transfection of hMSCs, cytotoxicity was measured by a PrestoBlue^®^ assay kit and protocol from Invitrogen™ (Cat# A13261). Briefly, the medium was aspirated, and each well was given a 100 µL of 10% Prestoblue^®^ prepared in the hMSC cell culture medium, and incubated for 1 h at 37 °C and 5% CO_2_. Thereafter, fluorescence was measured using a fluorescence spectrophotometer. The emission was measured at 590 nm and excitation was measured 560 nm. The background was subtracted from all the obtained values, and average values were plotted from three independent experiments. All experiments were done in triplicates and repeated thrice with similar results. Statistical analysis was done using one-way ANOVA with Bonferroni’s multiple comparison corrections (* *p* < 0.0001).

All concentrations of plasmid DNA and the respective ratios of Lipofectamine^TM^ 2000 reagent employed during different experimental conditions are provided in the Supplementary Materials as Table S1.

### 3.6. RNA Isolation and qRT-PCR. miRCURY^TM^

An RNA Isolation Kit (#300110) and protocol from EXIQON (Vedbaek, Denmark) were used for RNA extraction. Concisely, medium from the culture plates was aspirated and ice cold-PBS was employed for washing of cells twice. Afterward, direct 350 µL of lysis solution was added to each well of the 24-well cell culture plate. The culture plate was gently tapped and lysis buffer swirled around the well surface for 5 min to lyse the cells, and the resulting lysate was collected to a sterile Eppendorf tube. To this lysate-containing Eppendorf tube, 200 µL of 96–100% ethanol was added and vortexed for 10 s. Thereafter, the lysate was transferred to the RNA extraction column, followed by centrifugation at 14,000 rpm for 1 min. Thereafter, we performed column washing with the help of washing solution and consequent centrifugation at 14,000 rpm for 1 min. The steps of column washing were repeated for two more times and the flow through was discarded. Into a fresh 1.7 mL elution tube, the column was properly placed and 30 µL of the elution buffer was added to the column, and then centrifugation was performed for 2 min at 200 rpm and 1 min at 14,000 rpm to collect the RNA. To extract RNA from HA hydrogels (200 µL each), the medium was aspirated, each gel was washed twice with ice-cold PBS, and they were collected into 2 mL sterile and low protein binding Eppendorf tubes. Thereafter, each Eppendorf tube containing gel was given 350 µL of the RNA lysis buffer along with 200 µL 96–100% ethanol at 4 °C, followed by tissue lyser-assisted gel crushing and centrifugation at 14,000 rpm for 10 min at 4 °C. Thereafter, the supernatant was carefully collected to an RNA extraction column and followed further steps as explained above to extract RNA in 30 µL of the elution buffer. Subsequently, a High Capacity RNA to cDNA kit and protocol from Applied Biosystems (Foster City, CA, USA) was used for the preparation of cDNA, and qRT-PCR was performed with 500 ng cDNA with TaqMan^®^ Fast Universal PCR Master Mix (2×), no AmpErase^®^ UNG (Applied Biosystems) on MyiQ^TM^ Single color Real-Time PCR detection system from Bio-Rad (Hercules, CA, USA). BMP-2 primers (Hs00154192_m1), TBP primers (Hs00427620_m1), RUNX2 primers (Hs01047973_m1), SOX9 (Hs00165814_m1), ALPL (Hs01029144_m1), OPN (Hs00959010_m1), OCN (Hs01587814_g1), COL1A1 (Hs00164004_m1), COL2A1 (Hs00264051_m1) were obtained from Applied Biosystems (Cat # 4331182). Data from samples with a *C*t (Cycle threshold) value less than or equal to 35 were considered for further analysis. Relative quantification analysis was executed employing a comparative CT technique according to the manufacturer’s guidelines (Applied Biosystems). Statistical analysis was performed using one-way ANOVA with Bonferroni’s multiple comparison corrections (* *p* < 0.0001). Delta *C*t values (averaged from the technical replicates that were obtained by subtracting the house keeping gene Ct from target gene *C*t) were employed to assess the statistical significance. Gene expression values are stated as fold change relative to controls.

### 3.7. Western Blotting

After 48 h of transfection of cells in 2D, the medium was removed and the washing step was repeated twice with ice-cold PBS. Scraping of cells was performed and cells were collected at 1600 rpm for 5 min. Subsequently, 50 µL of the RIPA lysis buffer (Cat #R0278) with a 1% Protease Inhibitor cocktail from Sigma (Cat #P8340, Saint Louis, MO, USA) was given to each cell pellet. Pipetting up and down of the cell pellets were carried out until they were dissolved in the lysis solution, briefly vortexed, incubated on ice for 30 min and centrifuged at 14,000 rpm at 4 °C for 10 min. The supernatant was collected without disturbing the pellet. To isolate protein from HA hydrogels, the medium was aspirated, each gel was washed twice with ice-cold PBS, and they were (200 µL each) collected into 2 mL sterile low protein binding Eppendorf tubes. Thereafter, each Eppendorf tube containing gel was given 100 µL of the RIPA lysis buffer (Cat #R0278) with a 2% Protease Inhibitor cocktail from Sigma (Cat #P8340) at 4 °C and allowed them to quickly freeze in liquid nitrogen, followed by gel crushing using tissue lyser. Thereafter, we performed centrifugation at 14,000 rpm at 4 °C for 20 min and carefully collected supernatant. Protein concentrations were measured using the Bradford method. Thereafter, SDS-PAGE was used to separate 20 µg of soluble protein and was then transferred to polyvinylidene difluoride (PVDF) membrane (Millipore, Temecula, CA, USA). Primary antibodies raised in rabbit against BMP-2 (1:1000 dilution; Cat# ab14933; Abcam, Cambridge, MA, USA), Col1A1 (1:1000 dilution; ab-34710; Abcam), Col2A1 (1:1000 dilution; ab-34712; Abcam), and TBP (1:1000 dilution; Cat# 8515 Cell Signaling Technology, Beverly, MA, USA) were used to probe the protein bands. Anti-rabbit HRP-conjugated secondary antibodies (1:2000 dilutions; Cat# 170-6515, Bio-Rad) were used for the detection of primary antibodies, followed by target protein visualization with EMD Millipore Immobilon^TM^ Western Chemiluminescent HRP Substrate (ECL, Pierce, Thermo, USA). Images were acquired using Chemidoc^TM^ XRS + Systems from Bio-Rad. Relative quantification of western blot images was done with the help of ImageJ software (Version 1.49v, National Institutes of Health, Bethesda, MD, USA). Statistical analysis of the relative quantification of western blot images was performed using one-way ANOVA with Bonferroni’s multiple comparison corrections (* *p* < 0.00001).

## 4. Conclusions

In conclusion, we have developed an optimal method for ex vivo transfection of plasmid DNA in hMSCs followed by encapsulation in HA hydrogel. For successful gene therapy applications, it is necessary that reliable and clinically translatable methods of gene delivery be developed and evaluated for their efficacy and safety before translating into clinical practice. Our approach using suspension cells with 5-min incubation with lipoplexes offers enormous potential for developing such a clinically translatable platform that can be adopted for any cell-based therapies. Our data suggest that cationic lipids such as Lipofectamine^TM^ 2000 are toxic to cells and such toxicity could be partially eliminated by removing excess reagents by centrifugation. Transfection of hMSCs with BMP-2 plasmid demonstrated an osteogenic differentiation of stem cells with no sign of chondrogenic intermediate within our experimental time frame (21-days). We believe that such cell-laden hydrogels will pave the way for developing the next generation of osteo-inductive materials that will be safe, as it will allow autologous stem cell transplantation with patient-derived bone-marrow aspirate that could be transiently transfected and delivered in vivo using a biocompatible hydrogel. Such a strategy will also reduce the cost associated with conventional cell-based therapies.

## Supplementary Materials

Supplementary materials can be found at http://www.mdpi.com/1422-0067/20/1/56/s1.

## Abbreviations

MSC	Mesenchymal stromal/stem cells (MSCs)
rhBMP-2	Recombinant human bone morphogenetic protein-2
GAMs	Gene-activated 3D matrices
LPLD	Lipoprotein lipase deficiency
GAGs	Glycosaminoglycans
HA	Hyaluronic Acid
PVA	Polyvinyl alcohol
qRT-PCR	Quantitative real time polymerase chain reaction
2D	Two dimension
3D	Three dimension
RUNX2	Runt related transcription factor 2
SOX-9	SRY (Sex-Determining Region Y)-Box 9 Protein
