# Supplementary material for: Innovative Strategy for 3D Transfection of Primary Human Stem Cells with BMP-2 Expressing Plasmid DNA: A Clinically Translatable Strategy for Ex Vivo Gene Therapy
Source: Int J Mol Sci. 2018 Dec 23;20(1):56. doi: 10.3390/ijms20010056 (PMC6337215; doi:10.3390/ijms20010056)
Supplement: Supplementary file 1 [file ijms-20-00056-s001.pdf]

Supplementary Information

Innovative strategy for 3D transfection of primary human stem cells with BMP-2 expressing plasmid DNA: A clinically translatable strategy for bone regeneration

Maruthibabu Paidikondala[1], Sandeep Kadekar[1], Oommen P. Varghese[1]*

***Corresponding author**

1 Translational Chemical Biology Laboratory, Department of Chemistry, Ångström Laboratory, Uppsala University, 751 21, Uppsala, Sweden.

E-mail: oommen.varghese@kemi.uu.se

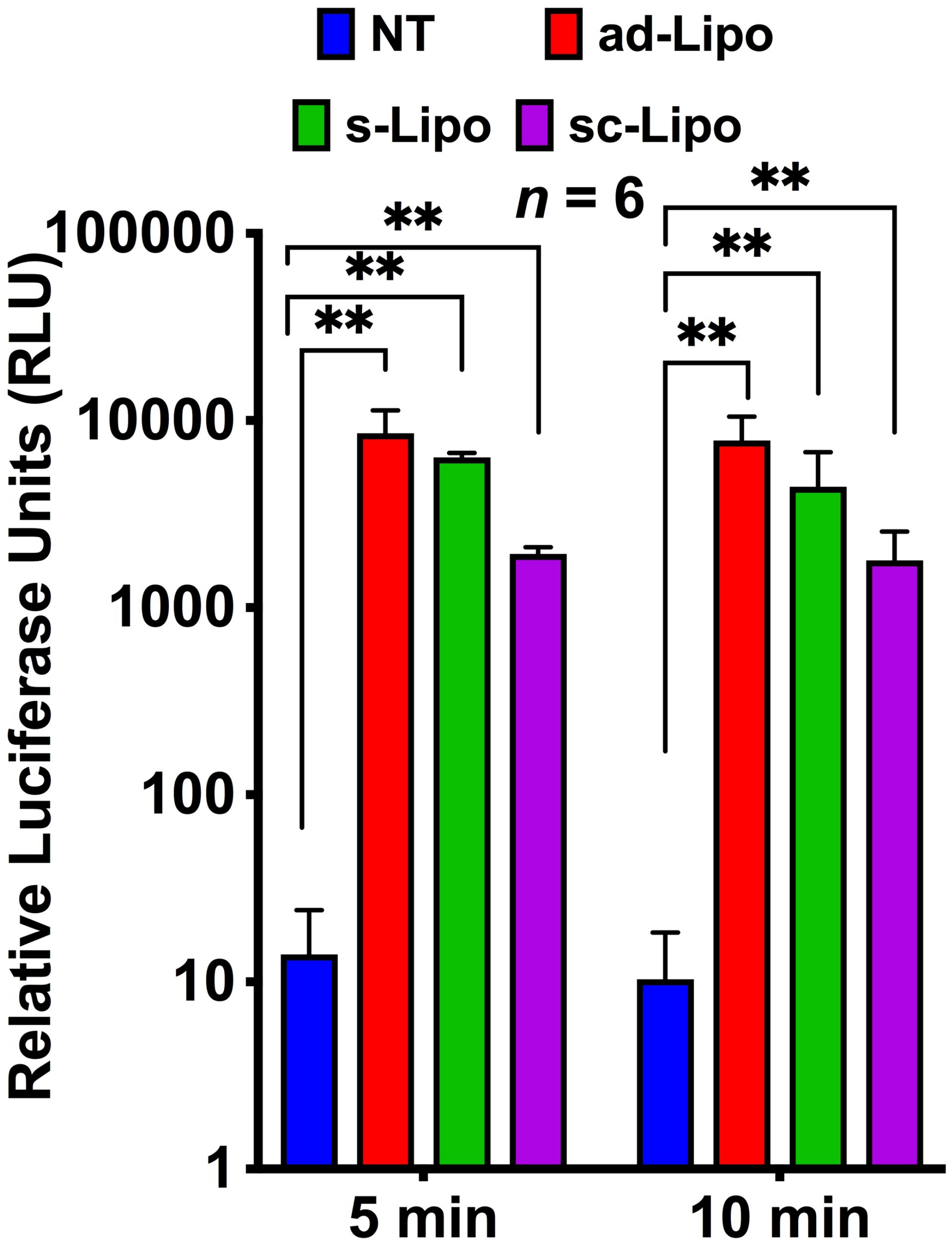

Fig. S1 Luciferase plasmid expression analysis using ONE-Glo™ Luciferase Assay System in hMSCs transfected with the assistance of Lipofectamine 2000 reagent in 2D. Mean values ± SD of 2 independent experiments done in triplicates ($n = 6$) are shown. Statistical analysis was performed using one-way ANOVA with Bonferroni's multiple comparison corrections (*$p < 0.0001$).

Transfection *condition*	Number of cells Per well/gel	Volume of Lipofectamine Per well/gel	Total amount of Plasmid DNA transferred
**Adherent (2D)**	**35000 cells per well in a 24-well plate**	**1.5 µL**	**100 ng**
**Suspension in 2D**	**35000 cells per well in a 24-well plate**	**1.5 µL**	**100 ng**
**Suspension followed by centrifugation in 2D**	**35000 cells per well in a 24-well plate**	**1.5 µL**	**100 ng**
**Suspension/direct addition in 3D** (gene expression analysis)	**200000 cells per 200 µL gel in a well of a 24-well plate**	**6 µL**	**500 ng**
**Suspension followed by centrifugation in 3D** (gene expression analysis)	**200000 cells per 200 µL gel in a well of a 24-well plate**	**6 µL**	**500 ng**
**Suspension/direct addition in 3D** (cell viability)	**100000 cells per 100 µL gel in a well of a 96-well plate**	**3 µL**	**250 ng**
**Suspension followed by centrifugation in 3D** (cell viability)	**100000 cells per 100 µL gel in a well of a 96-well plate**	**3 µL**	**250 ng**

Table S1. Table showing different volumes of Lipofectamine 2000 reagent, and concentrations of plasmid DNA used to transfect cells in different experimental conditions in 2D and 3D.
